# Efficacy and Safety of Steamed Ginger Extract for Body Weight and Body Fat Reduction in Overweight Adults: A Randomized, Double-Blind, Placebo-Controlled Clinical Trial

**DOI:** 10.3390/nu18020366

**Published:** 2026-01-22

**Authors:** Jeong Eun Kwon, Yeong-Geun Lee, Inhye Kim, Jaewoo Bae, Se-Chan Kang, Hyang-Im Baek

**Affiliations:** 1 Department of Oriental Medicine Biotechnology, Kyung Hee University, Yongin 17104, Republic of Korea; jjung1169@hanmail.net (J.E.K.); lyg629@nate.com (Y.-G.L.); 2 Integrated Metabolomics Research Group, Western Seoul Center for Korea Basic Science Institute, Seoul 03759, Republic of Korea; 3 Genencell Co., Ltd., Yongin 16950, Republic of Korea; inhyekim@genencell.co.kr; 4 3H LABS Research Institute, 3H LABS Co., Ltd., Goyang 10391, Republic of Korea; jerry@3h-labs.com; 5 Department of Food Science & Nutrition, Woosuk University, Wanju 55338, Republic of Korea

**Keywords:** steamed ginger extract, GGE03, 1-dehydro-6-gingerdione, obesity management, anti-obesity, overweight, body fat, body weight, metabolic health

## Abstract

Objective: This randomized, double-blind, placebo-controlled clinical trial assessed the efficacy and safety of steamed ginger extract (GGE03), standardized to high levels of 1-dehydro-6-gingerdione (GD), in reducing body fat and weight among overweight individuals. Methods: Eighty adults aged 18 to 60 years, with a body mass index (BMI) of 25.0 to 29.9 kg/m^2^, were randomly assigned to receive either GGE03 (*n* = 40; 480 mg/day) or a placebo (*n* = 40) for 12 weeks. Efficacy and safety parameters were evaluated at baseline and after the intervention period. Results: After 12 weeks, the GGE03 group showed statistically significant reductions in body fat percentage and body fat mass compared to the placebo group, as measured by dual-energy X-ray absorptiometry (DEXA). Additionally, significant decreases in body weight, BMI, waist circumference, and hip circumference were observed following GGE03 supplementation. Serum triglyceride (TG) and total cholesterol (TC) levels were also significantly lower in the GGE03 group compared to the placebo group. No product-related adverse events or clinically significant laboratory abnormalities were noted, indicating that GGE03 was well tolerated. Conclusions: Twelve weeks of GGE03 supplementation were associated with statistically significant improvements in body composition and lipid parameters without safety concerns. These findings support the potential of GD-standardized GGE03 as a well-tolerated functional dietary ingredient for body fat management and metabolic health.

## 1. Introduction

Obesity is a chronic, multifactorial disease characterized by abnormal or excessive fat accumulation resulting from an imbalance between energy intake and expenditure [1,2]. This imbalance results in excessive energy being stored as fat, which can lead to metabolic disturbances and various health complications [3]. The global prevalence of obesity is rising at an alarming rate, with approximately 1 billion people classified as overweight, and one-third of them considered obese, according to the World Health Organization (WHO) [4,5]. Beyond its impact on individual health, the socioeconomic burden of obesity has more than doubled over the past decade, increasing by 2.22 times—outpacing the growth rates associated with smoking (1.62 times) and alcohol consumption (1.56 times) [6]. This burden is expected to increase further in the future when intangible costs, such as reduced quality of life and psychosocial distress, are considered [7].

The condition arises from an imbalance between energy intake and expenditure, influenced by complex genetic and environmental factors, including neuroendocrine disruptions and metabolic disturbances. Contributing factors include irregular eating habits, excessive caloric intake, physical inactivity, endocrine disorders, genetic predispositions, psychological factors, and certain medications [8,9,10,11]. Obesity significantly raises the risk of developing metabolic and chronic diseases such as type 2 diabetes mellitus, cardiovascular disease, dyslipidemia, nonalcoholic fatty liver disease, hypertension, chronic kidney disease, and depression [12,13,14].

Approaches to weight management are multidisciplinary and include lifestyle modifications, pharmacological interventions, bariatric surgery, and the use of functional foods or nutraceuticals [15]. However, conventional anti-obesity drugs such as metformin, orlistat, lorcaserin, phentermine, topiramate, naltrexone, bupropion, and liraglutide are often associated with adverse effects, including nausea, constipation, hypoglycemia, and dyspepsia, limiting their long-term use [16,17]. Accordingly, there is increasing interest in developing safe and effective natural products as alternative therapies for obesity [18]. Functional ingredients derived from standardized natural extracts containing well-characterized bioactive compounds are promising candidates for managing obesity with minimal side effects. In this context, nutritional interventions targeting oxidative stress, chronic low-grade inflammation, and dysregulated lipid metabolism have gained attention as complementary approaches to obesity management [19,20,21]. Ginger (*Zingiber officinale*), known for its antioxidant and anti-inflammatory properties, has been investigated as a dietary intervention relevant to key mechanisms underlying obesity-related metabolic dysfunction [22,23,24].

Ginger is one of the most widely consumed spices worldwide and has long been used in both foods and traditional medicine for over two millennia [25,26]. Chemically, ginger contains a diverse spectrum of phenolic bioactive compounds, including gingerols, shogaols, paradols, and zingerone, which are collectively responsible for its antioxidant, anti-inflammatory, antimicrobial, anticancer, neuroprotective, cardiovascular protective, respiratory protective, anti-obesity, antidiabetic, antinausea, and antiemetic activities [22,27,28,29]. However, several ginger-derived constituents are chemically unstable and susceptible to degradation during processing and storage, resulting in substantial variability in phytochemical composition and biological efficacy among ginger preparations [30,31,32].

To overcome these limitations, thermal processing techniques, including steam processing, have been explored to improve the stability and reproducibility of ginger bioactives [33,34,35]. Among these approaches, steam processing—a high-temperature, high-pressure treatment—has received increasing attention because it induces marked alterations in the phytochemical composition of ginger, which may contribute to enhanced biological activity [33].

The steamed ginger extract (GGE03) produced through this process is standardized to high levels of 1-dehydro-6-gingerdione (GD), a thermally stable bioactive compound that is rarely found in raw ginger but is markedly increased during steam treatment [35]. Experimental studies have shown that GD exhibits antioxidant and lipid-modulating activities, which are closely associated with the regulation of metabolic pathways involved in lipid and glucose homeostasis [35,36,37]. Additionally, GGE03 has demonstrated lower toxicity and greater overall biological efficacy in preclinical studies [35,36,37]. Collectively, steamed ginger extract has shown a range of biological functions, including antioxidant and anti-inflammatory effects. These properties contribute to gastric protection [36,37,38], anti-osteoarthritis activity [39], anti-obesity effects [34,40,41,42,43,44], anti-diabetic properties [35], and anticancer activity [33].

Preclinical studies using the identical extract have demonstrated that GGE03 exerts anti-obesity and metabolic regulatory effects through coordinated modulation of lipid and glucose metabolism. In high-fat diet–fed animal models, GGE03 significantly reduced body weight gain and adiposity while improving serum lipid profiles and hepatic steatosis [34,41]. Mechanistically, these effects were associated with activation of AMP-activated protein kinase (AMPK) and subsequent suppression of lipogenic and adipogenic pathways, including downregulation of sterol regulatory element-binding protein-1c (SREBP-1c), fatty acid synthase (FAS), acetyl-CoA carboxylase (ACC), and peroxisome proliferator-activated receptor-γ (PPARγ) [34,41]. In parallel, GGE03 enhanced glucose utilization in skeletal muscle cells through upregulation of insulin receptor substrate-1 (IRS-1) and glucose transporter type 4 (GLUT4), suggesting improved peripheral insulin sensitivity [41].

Other natural resources with metabolic benefits have also been investigated, such as turmeric (*Curcuma longa*) and its principal constituent curcumin, which have been reported to improve inflammatory status and certain obesity-related indices primarily through anti-inflammatory pathways [45,46]. However, curcumin is well known to exhibit low oral bioavailability due to limited intestinal absorption and extensive first-pass metabolism [47,48]. In contrast, steamed ginger extract standardized to GD represents a chemically stable botanical ingredient with demonstrated metabolic relevance, although the mechanistic evidence supporting these effects is primarily derived from preclinical models.

Although preclinical evidence indicates that GD-standardized GGE03 may influence adiposity and lipid-related metabolic pathways, previous human intervention studies of ginger-based preparations have reported inconsistent findings, with variable and often limited effects on body composition and metabolic parameters. To address this gap, a randomized, double-blind, placebo-controlled clinical trial was conducted to evaluate the efficacy and safety of GGE03 on body composition, lipid parameters, and anthropometric measures in overweight adults.

## 2. Materials and Methods

### 2.1. Ethics Statement

The research protocol (Protocol No. FF22-1H1), together with the informed consent materials, received ethical approval from the Santosh Hospital Institutional Ethics Committee (IRB No. SHIEC/CC/FEB_2022/01) on 17 January 2022. This study was registered with the Clinical Trials Registry India (CTRI) on 4 February 2022 (CTRI number: CTRI/2022/02/040052). All participants voluntarily provided written informed consent before participating in the study. Ethical conduct of the study was ensured through adherence to the Declaration of Helsinki and the Good Clinical Practice (GCP) guidelines established by the International Council for Harmonisation (ICH). The design, conduct, and reporting of this trial adhered to the Consolidated Standards of Reporting Trials (CONSORT) recommendations.

### 2.2. Study Design

This single-center, 12-week, randomized, double-blind, placebo-controlled clinical trial was conducted at Santosh Hospital in Bangalore, India, from March 2022 to January 2023. The 12-week intervention duration was selected based on previous nutritional intervention studies in which similar timeframes have been commonly used to evaluate changes in body composition and lipid-related parameters.

Following a screening period of up to two weeks (Visit 0), participants who satisfied all predefined inclusion and exclusion criteria were enrolled and completed baseline evaluations. Eighty eligible participants were then allocated in a 1:1 ratio to receive either GGE03 (*n* = 40) or placebo (*n* = 40). The randomization sequence was generated using computer-based random number generation with CRAN R software (version 4.1.0; R Foundation for Statistical Computing, Vienna, Austria). Blinding was maintained throughout the trial, with both participants and investigators unaware of group assignments. Participants attended four visits during the 12-week period (Visit 1: week −2; Visit 2: week 0; Visit 3: week 6; Visit 4: week 12).

Dietary habits and physical activity levels were assessed at baseline and at the end of the intervention using standardized questionnaires. Participants were instructed to maintain their usual eating patterns, daily food intake, and regular physical activity routines throughout the study to minimize potential confounding effects on body composition outcomes.

### 2.3. Study Participants

Eighty overweight adults participated in this study. The inclusion criteria were: (1) men and women aged 18 to 60 years; (2) a body mass index (BMI) between 25.00 and 29.99 kg/m^2^, indicating the overweight range; and (3) voluntary provision of written informed consent prior to participation.

The exclusion criteria included: (1) a known history of hypersensitivity to herbal extracts or dietary supplements; (2) participation in a commercial weight loss program or clinical trial for obesity/overweight within the last three months; (3) intentional weight loss of at least 5% within three months prior to screening; (4) any weight loss-related surgery, such as gastroplasty or intestinal resection, within six months prior to screening; (5) consumption of drugs or diet foods that affect weight within three months prior to screening; (6) a significant psychological medical history or current disease (e.g., schizophrenia, epilepsy, anorexia, bulimia) or a history of substance abuse; (7) severe cerebrovascular disease (e.g., cerebral infarction, cerebral hemorrhage), heart disease (e.g., angina pectoris, myocardial infarction, heart failure, or treatment-required arrhythmia), or lung disease (e.g., chronic obstructive pulmonary disease) within the last six months; (8) endocrine diseases such as hypothyroidism, polycystic ovary syndrome, or Cushing’s syndrome; (9) fasting blood glucose levels ≥ 126 mg/dL, random blood glucose levels ≥ 200 mg/dL, or diabetic patients taking oral hypoglycemic agents or insulin; (10) severe liver dysfunction (alanine and aspartate aminotransferase levels 2.5 times the upper limit of normal) or kidney dysfunction (creatinine > 2.0 mg/dL); (11) signs or symptoms of severe gastrointestinal issues, such as heartburn or indigestion; (12) use of drugs (e.g., absorbent laxatives, appetite suppressants, health supplements for obesity, psychotropic medications such as antidepressants, beta-blockers, diuretics, contraceptives, steroids, and female hormones) affecting body weight within the last month; (13) inability to exercise due to musculoskeletal disease; (14) females who are breastfeeding, pregnant, or planning to become pregnant during the study period; or (15) any condition that, in the investigator’s opinion, rendered the individual ineligible for this clinical trial.

### 2.4. Study Products and Interventions

The investigational products evaluated in this trial were supplied by Genencell Co., Ltd. (Yongin, Republic of Korea). GGE03 was manufactured according to a standardized process described in previous studies [34,36,37,38,39,41]. In brief, ginger was washed three times with distilled water, steamed at 97 °C for 2 h under a pressure of 2–2.5 kgf cm^−2^, and then dried at 50 °C for 30 h. GGE03 was produced by extracting the steamed ginger with 15-fold 70% ethanol (*v*/*v*) for 15 h at 85 °C. The extract was filtered and concentrated through vacuum evaporation, and the resulting concentrate was spray-dried create obtain a final powdered product containing less than 5% moisture. GD content in GGE03 was standardized to 1.15 mg/g.

Participants were randomly assigned to receive either GGE03 or a placebo for 12 weeks. Participants in the active group received GGE03 at a daily dose of 480 mg, administered orally as two tablets per day. The placebo tablets contained only microcrystalline cellulose, an inert excipient with no known effects on body weight or metabolic parameters. Both the placebo and active tablets were manufactured to be indistinguishable in appearance, weight, and taste to ensure blinding.

### 2.5. Efficacy Outcome Measures

Efficacy evaluations were performed at baseline and after 12 weeks of intervention, with the same measurement instruments used at all time points to maintain consistency. The primary efficacy outcomes measured were body fat percentage and body fat mass, while secondary outcomes included anthropometric parameters such as body weight, BMI, waist circumference, hip circumference, and waist-to-hip ratio (WHR), as well as lean body mass and blood biomarkers including total cholesterol (TC), triglycerides (TG), high-density lipoprotein cholesterol (HDL-C), low-density lipoprotein cholesterol (LDL-C), and adiponectin. The primary outcome was prespecified, and secondary outcomes were included as supportive measures.

Body fat percentage, body fat mass, and lean body mass were assessed using dual-energy X-ray absorptiometry (DEXA; Lunar DPX NT, GE Healthcare, Madison, WI, USA). BMI was calculated by dividing body weight (kg) by height squared (m^2^), and WHR was determined by the ratio of waist circumference (cm) to hip circumference (cm). Blood samples were collected following an overnight fast.

### 2.6. Safety Outcome Measures

Safety evaluations were conducted for all participants who received at least one dose of the study product. The safety outcomes assessed included the incidence of adverse events (AEs), vital signs, hematology, blood biochemistry, and urinalysis.

AEs were monitored continuously throughout the study and documented at each visit based on participant reports and investigator observations. Vital signs, such as systolic and diastolic blood pressure (SBP, DBP), pulse rate, and body temperature, were measured at every visit. Hematological parameters included white blood cell (WBC), red blood cell (RBC), hemoglobin, hematocrit, and platelet counts. Blood chemistry analysis included aspartate aminotransferase (AST), alanine aminotransferase (ALT), alkaline phosphatase (ALP), gamma-glutamyl transferase (GGT), albumin, total protein, total bilirubin, glucose, blood urea nitrogen (BUN), creatinine, and uric acid levels. Urinalysis assessed pH and specific gravity. All laboratory analyses were performed following a 12 h overnight fast at both baseline and at the completion of the 12-week intervention period.

### 2.7. Statistical Analysis

This study aimed to evaluate the efficacy of 12 weeks of GGE03 supplementation in reducing body fat percentage compared to a placebo. The required sample size was estimated based on a previous clinical study with a similar design and primary endpoint [49]. For this calculation, a two-sided test was employed with a power of 80% and a significance level of 5%. The sample size for each group was adjusted for a 20% dropout rate, resulting in a final sample size of 40 subjects per group, totaling 80 participants.

Statistical analyses were performed using the Statistical Package for the Social Sciences (SPSS version 22.0; Chicago, IL, USA). Continuous data are reported as means with standard deviations, whereas categorical data are presented as frequencies and percentages.

Statistical analyses were conducted according to the prespecified study protocol. Efficacy analyses were performed in the per-protocol set (PPS), defined as participants who completed the study with at least 80% compliance, had available post-randomization data for the primary efficacy outcomes, and did not experience any major protocol deviations. This analysis set was selected to assess the effects of the intervention under controlled conditions, with primary emphasis placed on the predefined primary endpoint.

Within-group comparisons were conducted using a paired *t*-test or the Wilcoxon signed-rank test, selected according to the normality of the data. For comparisons of changes from baseline between groups, either an independent *t*-test or the Wilcoxon rank-sum test was applied, depending on the underlying data distribution. Statistical significance was defined as a *p*-value of less than 0.05.

## 3. Results

### 3.1. Participant Characteristics

**Figure 1:**
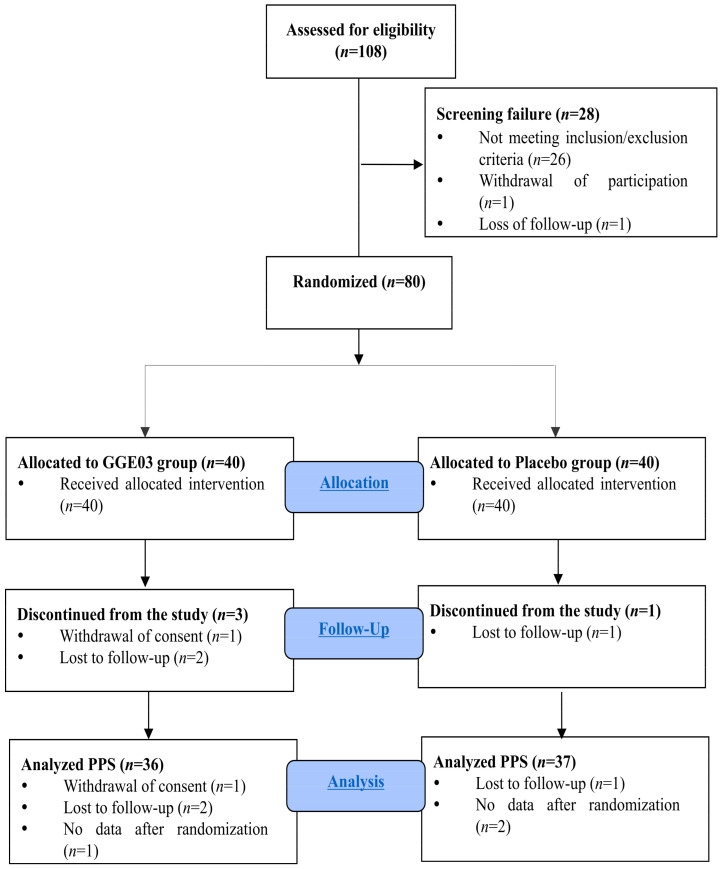
Flow diagram of the study participants.

A total of 108 volunteers were initially screened for eligibility. Among them, 28 participants were excluded prior to randomization for the following reasons: they did not meet the inclusion or exclusion criteria (*n* = 26), withdrew consent (*n* = 1), or were lost to follow-up during the screening period (*n* = 1). The remaining 80 participants were randomly assigned to either the GGE03 group (*n* = 40) or the placebo group (*n* = 40). During the 12-week intervention period, three participants in the GGE03 group and one participant in the placebo group discontinued the study. In the GGE03 group, one participant withdrew consent, and two participants were lost to follow-up due to failure to respond. In the placebo group, one participant was lost to follow-up for the same reason. Consequently, 37 participants in the GGE03 group and 39 participants in the placebo group completed the study. For the efficacy analysis based on the PPS, participants for whom the primary efficacy outcome was not assessed after randomization were excluded. This resulted in the exclusion of one participant in the GGE03 group and two participants in the placebo group, leaving 36 participants in the GGE03 group and 37 participants in the placebo group included in the PPS analysis. Figure 1 presents the overall process of participant enrollment, group allocation, follow-up, and data analysis.

Product intake compliance was high in both groups, with an average exceeding 90% throughout the intervention period. There were no significant differences in compliance rates between the GGE03 and placebo groups (*p* > 0.05), and no participants were withdrawn due to poor adherence to product intake.

Table 1 summarizes the baseline demographic characteristics of the participants. There were no significant differences between the GGE03 and placebo groups in general baseline variables, including sex, age, height, weight, BMI, body fat percentage, body fat mass, marital status, current drinking, and current smoking status (*p* > 0.05).

### 3.2. Efficacy Outcomes

**Figure 2:**
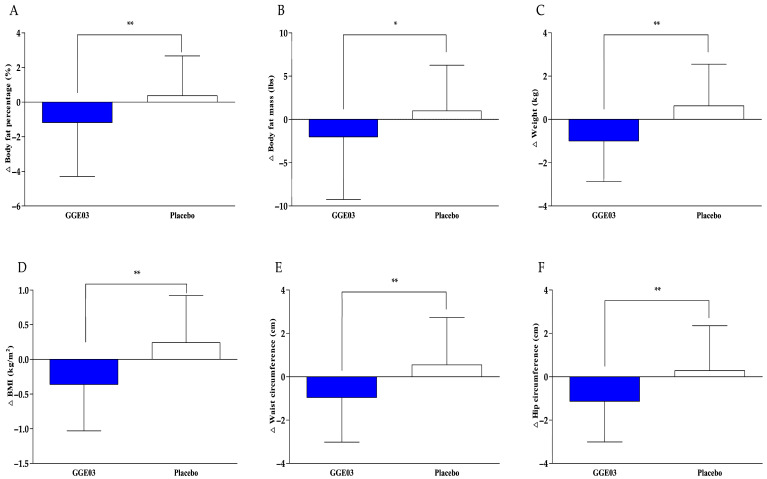
Changes in body composition (DEXA) and anthropometric parameters after 12-week intervention. (**A**) Changes in body fat percentage, (**B**) body fat mass, (**C**) weight, (**D**) BMI, (**E**) waist circumference, and (**F**) hip circumference in the GGE03 and placebo groups. Values are presented as mean ± SD. Analyzed by Wilcoxon rank sum test for change value between the groups (GGE03 vs. placebo). Δ indicates the change from baseline. * *p* < 0.05, ** *p* < 0.01 vs. placebo group.

Efficacy biomarkers were measured before the intervention and again 12 weeks after it. Body composition parameters assessed by DEXA, including body fat percentage, body fat mass, and lean body mass, are presented in Table 2 and illustrated in Figure 2. The primary efficacy endpoint, body fat percentage, showed a significant decrease after 12 weeks of GGE03 supplementation compared to baseline (mean change: −1.18 ± 3.12%, *p* = 0.028), while no significant change was noted in the placebo group (0.37 ± 2.29%, *p* > 0.05). Between-group comparisons of the changes from baseline indicated a statistically significant difference favoring the GGE03 group (*p* = 0.009). Additionally, body fat mass decreased by −2.01 ± 7.25 lbs in the GGE03 group, whereas it increased by 0.98 ± 5.28 lbs in the placebo group. Although within-group changes were not statistically significant in either group (*p* > 0.05), the difference in change between groups was statistically significant (*p* = 0.034). Lean body mass did not differ significantly between the groups, suggesting that GGE03 supplementation decreased body fat without adverse effects on lean tissue.

Anthropometric measures such as body weight, BMI, waist and hip circumferences, and WHR are summarized in Table 3 and depicted in Figure 2. After 12 weeks of intervention, the GGE03 group showed a significant reduction in body weight compared to baseline (−1.00 ± 1.88 kg, *p* = 0.007), while the placebo group showed a nonsignificant increase (0.63 ± 1.92 kg, *p* = 0.090). The comparison between groups revealed a statistically significant difference favoring the GGE03 group (*p* = 0.001). Additionally, BMI in the GGE03 group significantly decreased (−0.36 ± 0.67 kg/m^2^, *p* = 0.006), whereas the placebo group experienced a significant increase (0.24 ± 0.68 kg/m^2^, *p* = 0.046). The difference between the two groups was significant (*p* = 0.001), indicating that GGE03 supplementation resulted in a greater reduction in BMI compared to the placebo. A significant decrease in waist circumference was observed in the GGE03 group (−0.96 ± 2.06 cm, *p* = 0.008), while the placebo group showed a significant increase (0.55 ± 2.18 cm, *p* = 0.038). The reduction in waist circumference was significantly greater in the GGE03 group than in the placebo group (*p* = 0.001). Hip circumference also decreased significantly in the GGE03 group after the 12-week intervention (−1.13 ± 1.88 cm, *p* = 0.001), while the placebo group showed no significant change (0.28 ± 2.07 cm, *p* = 0.172). The difference in change values between the groups was significant (*p* = 0.001). However, there were no significant changes in WHR within or between groups during the study period (*p* > 0.05).

**Figure 3:**
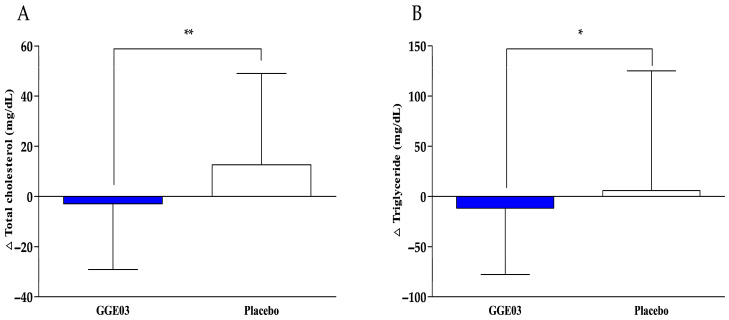
Changes in serum lipid parameters after 12-week intervention. (**A**) Changes in total cholesterol, and (**B**) triglycerides in the GGE03 and placebo groups. Values are presented as mean ± SD. Analyzed by Wilcoxon rank sum test for change value between the groups (GGE03 vs. placebo). Δ indicates the change from baseline. * *p* < 0.05, ** *p* < 0.01 vs. placebo group.

Table 4 summarizes serum lipid parameters, including TC, TG, LDL-C, HDL-C, and adiponectin levels, which are illustrated in Figure 3. After 12 weeks of intervention, TC slightly decreased in the GGE03 group (−2.94 ± 26.18 mg/dL, *p* = 0.05), while it significantly increased in the placebo group (12.62 ± 36.35 mg/dL, *p* = 0.042). The between-group comparison revealed a significant difference (*p* = 0.007), indicating that GGE03 supplementation effectively prevented the increase in TC seen in the placebo group. Serum TG levels significantly decreased in the GGE03 group (−11.72 ± 66.14 mg/dL, *p* = 0.007), while no significant change was observed in the placebo group (5.81 ± 119.36 mg/dL, *p* = 0.934). The difference between the groups was statistically significant (*p* = 0.043), suggesting that GGE03 intake played a role in reducing TG levels. In contrast, no significant differences were found in LDL-cholesterol, HDL-cholesterol, or adiponectin levels between the groups (*p* > 0.05).

Throughout the 12-week study, there were no significant differences in dietary intake or physical activity between the GGE03 and placebo groups (*p* > 0.05), indicating that the observed efficacy outcomes were not influenced by lifestyle factors.

### 3.3. Safety Outcomes

Safety was evaluated based on AEs, laboratory tests, and vital signs, with detailed results summarized in Table 5.

During the study period, adverse events were reported by eight participants (six in the GGE03 group and two in the placebo group), with no significant difference in incidence between groups (*p* > 0.05). All reported AEs were mild in intensity, no serious adverse events occurred, and none were considered related to the investigational product.

Laboratory safety parameters, including complete blood count (CBC), biochemical, and urinalysis measures, remained within normal reference ranges throughout the study, with no clinically meaningful differences between groups. Vital signs, including blood pressure and pulse rate, remained stable over the 12-week intervention period. Overall, GGE03 supplementation was well tolerated, with no safety concerns identified.

## 4. Discussion

This study is, to our knowledge, the first randomized, double-blind, placebo-controlled clinical trial to assess the efficacy and safety of GGE03 standardized to high levels of GD in overweight adults. The 12-week supplementation with GGE03 resulted in statistically significant reductions in body fat percentage, total body fat mass, body weight, BMI, waist circumference, and hip circumference. Additionally, there were notable decreases in serum TC and TG levels compared to the placebo group. Throughout the intervention, no product-related adverse events or abnormal clinical findings were reported, indicating that GGE03 was well tolerated during the study period. These results provide clinical evidence that GD-standardized GGE03 is associated with improvements in body composition and lipid parameters in overweight individuals.

Previous human trials have examined the metabolic effects of ginger; however, their results have been inconsistent, primarily due to variations in extraction methods, marker compounds, and bioactive stability. Among these, Park et al. (2020) [40] conducted a randomized, double-blind, placebo-controlled clinical trial involving 80 overweight adults over a period of 12 weeks. They used a steamed ginger ethanolic extract standardized to 6-shogaol (5.89–8.83 mg/g) and reported significant reductions in body weight, BMI, and arm fat mass. However, changes in total body fat and abdominal fat were not statistically significant. These limited effects may, at least in part, be attributed to the thermolabile nature of 6-shogaol, which degrades during extraction and storage, leading to variability in its biological potency. In contrast, the present study used GGE03, a steamed ginger extract standardized to GD, which is a thermally stable and metabolically active marker compound produced through controlled steam processing [35,41]. GD exhibits greater chemical stability and stronger AMPK-activating, lipolytic, and anti-inflammatory properties than 6-shogaol [34,35]. This compositional advancement likely contributed to the systemic reductions in adiposity and lipid parameters observed in this study, which included total fat mass, BMI, and central obesity indices such as waist and hip circumferences. Notably, these changes were observed within a relatively short 12-week intervention period and across multiple related endpoints, supporting the biological relevance of the observed effects despite the limited study duration.

The between-group difference in percent body fat observed in this study (−1.55% points relative to placebo) is consistent with the pooled weighted mean differences reported in recent meta-analyses of randomized controlled trials of ginger supplementation. Specifically, systematic reviews have reported weighted mean differences ranging from—0.87% to −1.49% in percent body fat compared with control groups [50,51]. Because weighted mean differences represent net effects relative to placebo, these findings indicate that the magnitude of fat reduction observed with GGE03 falls within the reproducible range of effects reported for ginger-derived interventions. From a clinical standpoint, evidence from prior studies indicates that reductions in adiposity of this magnitude can translate into favorable changes in metabolic risk markers and adipose tissue function in overweight adults [52]. In addition, reductions in body weight and waist circumference, when observed alongside consistent decreases in body fat and lipid-related parameters, are commonly interpreted as indicators of improved central adiposity and metabolic risk [53,54].

Although previous randomized controlled trials [55] and meta-analyses [50,51,56] of ginger supplementation have reported beneficial effects on body weight and lipid metabolism, the magnitude and consistency of these effects have varied across studies, largely due to differences in extraction methods, marker compounds, and formulation characteristics. Therefore, the current trial provides stronger evidence that thermal conversion and GD-based standardization enhance both the chemical consistency and metabolic efficacy of ginger in human applications.

The reductions in body fat and serum lipids observed after GGE03 supplementation align with the established metabolic mechanisms of steamed ginger and its active compounds. Steaming ginger transforms unstable shogaol into more stable and bioactive derivatives, particularly GD, which enhances both their chemical stability and physiological effectiveness [36,37]. GD has been shown to have superior antioxidant, anti-inflammatory, and lipolytic activities compared to unprocessed ginger components. Experimental studies indicate that GD and GD-enriched steamed ginger extracts increase AMPK phosphorylation, enhance mitochondrial fatty acid oxidation, and suppress adipogenic transcription factors like PPAR-γ and C/EBPα, resulting in reduced lipid accumulation in adipocytes [34,41]. Furthermore, GD enhances insulinotropic activity by closing ATP-sensitive potassium (K_ATP) channels in pancreatic β-cells, which contributes to improved glucose utilization and energy metabolism [35]. Such multi-targeted metabolic modulation—encompassing fatty acid oxidation, improved insulin sensitivity, and reduced inflammation—likely explains the observed decreases in body fat, BMI, and serum lipids in the present study. Heat processing enhances the bioavailability and stability of phenolic compounds while reducing irritant components like 6-gingerol, thereby improving both efficacy and gastrointestinal tolerability [12,13]. In summary, the enhanced biological efficacy of GGE03 compared to conventional ginger extracts can be attributed to the thermal conversion process, which increases GD concentration, boosts AMPK-mediated fat oxidation, and stabilizes antioxidant and anti-inflammatory components. These mechanisms collectively account for the systemic reductions in adiposity and lipid parameters observed in this clinical trial.

Although these mechanistic findings are largely derived from in vitro and animal models, they provide a plausible biological context for interpreting the clinical outcomes observed in the present study, particularly the reductions in total fat mass, central adiposity, and serum TG. Moreover, the metabolic influence of GGE03 was reflected in selected serum lipid parameters. Alongside changes in body composition, GGE03 supplementation was associated with statistically significant reductions in serum TC and TG levels. Elevated TC and TG are key risk factors for atherosclerosis and metabolic syndrome, often associated with increased adiposity and insulin resistance [57]. The observed lipid-related changes are consistent with preclinical evidence showing that steamed ginger extract and GD-enriched fractions modulate pathways involved in hepatic lipid synthesis and fatty acid oxidation, including AMPK-related signaling [34,41,43]. Although HDL-C and LDL-C levels did not exhibit significant differences between groups, this may be explained by the relatively normal baseline lipid profiles of the participants and the limited intervention duration, as remodeling of lipoprotein fractions often requires longer exposure. Taken together, these findings may help to explain the observed reductions in serum TC and TG, suggesting that GGE03 supplementation is associated with short-term improvements in selected lipid-related parameters alongside reductions in adiposity.

Building on these mechanistic insights, the observed changes in anthropometric and body composition parameters further support the metabolic effects of GGE03 supplementation.

Body weight and BMI are key indicators of obesity and overall energy balance. In this study, both parameters significantly decreased after 12 weeks of GGE03 supplementation compared to baseline and the placebo group, highlighting GGE03’s effectiveness in promoting weight loss. The reductions in weight and BMI were accompanied by notable decreases in body fat percentage and fat mass, as assessed by DEXA, indicating that the weight loss primarily resulted from fat reduction rather than fluid loss or depletion of lean tissue. Clinically, even modest reductions in BMI are known to enhance metabolic risk factors, including insulin sensitivity, lipid profiles, and inflammatory markers [58,59]. Therefore, the concurrent improvements in BMI and body composition observed in this study further support the metabolic benefits of GGE03 supplementation in overweight adults.

Waist and hip circumferences are commonly used anthropometric indicators that reflect the distribution of abdominal and gluteofemoral fat, which are closely linked to metabolic and cardiovascular risk [60]. In this study, we found that both waist and hip circumferences significantly decreased after 12 weeks of GGE03 supplementation, indicating an improvement in body fat distribution. The reduction in waist circumference is especially noteworthy, as it signifies a decrease in visceral fat, which is closely associated with insulin resistance, dyslipidemia, and systemic inflammation [61,62]. The simultaneous reduction in hip circumference indicates a general decrease in subcutaneous fat. Together, these findings suggest that GGE03 not only reduced total body fat but also positively influenced anthropometric measures related to metabolic health.

Body composition was evaluated using DEXA, the gold standard for distinguishing between bone, fat, and lean tissue based on the differential attenuation of two X-ray energies [63]. DEXA offers superior accuracy and reproducibility compared to other noninvasive methods and is widely used in clinical research to detect changes in body fat [64]. In the present study, DEXA analysis revealed a significant reduction in both total body fat percentage and total fat mass after 12 weeks of GGE03 supplementation, confirming its effectiveness in reducing adiposity. These results, obtained through a validated and reliable measurement technique, provide strong evidence that GGE03 effectively decreases body fat in overweight adults.

Importantly, the DEXA analysis indicated that lean body mass (LBM) remained stable throughout the intervention period. This suggests that GGE03 facilitated selective fat reduction rather than overall weight loss. In contrast, weight loss accompanied by LBM loss, which is often seen in some dietary interventions, can lead to decreased resting energy expenditure, increased fatigue, reduced muscle strength, and a higher risk of injury [65]. Moreover, loss of LBM can lead to a metabolic slowdown, which may predispose individuals to regain weight [66]. LBM is crucial for sustainable weight management and preventing negative metabolic outcomes. In this context, the current findings indicate that GGE03 may be an effective strategy for improving body composition and maintaining weight loss while preserving lean tissue.

These findings collectively highlight the selective effects of GGE03 on body composition. The concurrent reductions in body fat mass, body fat percentage, BMI, waist and hip circumferences, along with improvements in serum TC and TG, suggest that GGE03 is associated with short-term improvements in body composition and lipid-related outcomes under controlled conditions. These findings are clinically meaningful because central adiposity and dyslipidemia are well-established risk factors for cardiometabolic disorders, such as insulin resistance, type 2 diabetes, and atherosclerotic cardiovascular disease [61,67]. GGE03 may help prevent metabolic disorders early in overweight individuals by addressing both adiposity and lipid dysregulation.

Regarding safety, ginger has a long-standing history as both a food and medicinal herb and is widely consumed with a favorable safety profile. The U.S. Food and Drug Administration (FDA) has classified ginger as “Generally Recognized As Safe” (GRAS) for use as a food ingredient [68]. Moreover, ginger has an excellent safety profile, with very few reported herb-drug interactions [69,70]. Consistent with these findings, previous clinical trials that administered GGE03 at a daily dose of 480 mg for 12 weeks to subjects with functional dyspepsia [38] and mild osteoarthritis [39] also reported no clinically meaningful changes in safety parameters. Regarding glucose-related parameters, although minor fluctuations were observed during the intervention period, these values remained within the normal physiological range and were not considered clinically meaningful. Such modest variations in fasting glucose have been frequently reported in nutritional intervention studies and are generally attributed to inter-individual variability and baseline metabolic status, rather than adverse metabolic effects of the intervention [71,72,73]. This aligns with the results of the current study. Therefore, daily supplementation with GGE03 was well tolerated over the study period, with no clinically relevant safety concerns observed.

Several limitations of this study should be acknowledged. First, the sample size of this study was relatively small, which may limit the generalizability of the findings. However, the number of participants was determined based on previous clinical trials with similar study designs and intervention durations, and the present study was adequately powered to detect short-term changes in body fat and body weight following GGE03 supplementation. Nevertheless, larger-scale studies are warranted to further confirm these findings and to extend their applicability to broader populations. In addition, the single-center design and the relatively homogeneous characteristics of the study population may further limit the generalizability of the findings to broader or more diverse populations. Second, the intervention period of this study was limited to 12 weeks, which allows evaluation of short-term effects of GGE03 supplementation on body composition and metabolic parameters but does not permit assessment of long-term efficacy or safety. Future studies with longer intervention periods are needed to determine whether the observed effects can be sustained over time and to further evaluate long-term outcomes. Third, although dual-energy X-ray absorptiometry (DEXA) was used to provide quantitative assessment of body composition, this method primarily reflects total body fat and does not distinguish between visceral and subcutaneous fat depots. Future studies incorporating imaging techniques such as computed tomography (CT) or magnetic resonance imaging (MRI) may help to further clarify regional fat distribution. In addition, although participants were instructed to maintain their usual diet and physical activity throughout the study period, unmeasured lifestyle factors may have influenced the outcomes. Finally, efficacy analyses were conducted using a per-protocol population, which should be considered when interpreting the findings. Third, although DEXA was used to enhance the accuracy of body composition assessment, it only provided an estimation of total body fat. Future studies that incorporate imaging techniques such as computed tomography (CT) could clarify GGE03’s effects on visceral and subcutaneous fat distribution. Additionally, although participants were instructed to maintain their usual diet and physical activity, residual lifestyle variations cannot be completely excluded.

Despite these limitations, the present study has notable strengths. It is the first randomized, double-blind, placebo-controlled clinical trial to evaluate a GD-standardized steamed ginger extract in humans, utilizing validated and quantitative endpoints, including DEXA-measured body composition and serum lipid biomarkers. The study demonstrated consistent and statistically significant changes in body fat, anthropometric indices, and lipid parameters without no product-related safety concerns.

## 5. Conclusions

In conclusion, a 12-week supplementation with GGE03, a steamed ginger extract standardized to high levels of GD, resulted in significant reductions in body fat percentage, body fat mass, body weight, BMI, waist circumference, hip circumference, and serum TC and TG levels compared to placebo, without any adverse effects related to the product. These findings indicate that GGE03 is associated with improvements in body composition and lipid parameters. Collectively, its favorable tolerability and standardized GD composition support the potential of GGE03 as a functional ingredient for body fat management and metabolic health. Further studies may help to better define its long-term benefits and broader applicability.

## Figures and Tables

**Table 1:** Baseline characteristics of the participants.

Characteristics	GGE03 Group (*n* = 36)	Placebo Group (*n* = 37)	*p*-Value ^1^ (Between Group)
Sex (M/F)	33/3	34/3	0.972 ^2^
Age (years)	36.39 ± 5.44	35.86 ± 7.33	0.729
Height (cm)	168.01 ± 7.91	167.31 ± 6.82	0.494 ^3^
Weight (kg)	75.00 ± 7.14	76.09 ± 7.57	0.530
BMI (kg/m^2^)	26.56 ± 1.31	27.14 ± 1.63	0.174 ^3^
Body fat percentage (%)	32.16 ± 6.99	32.38 ± 7.13	0.912 ^3^
Body fat mass (lbs)	52.74 ± 15.52	52.39 ± 14.28	0.919
Married (*n*, %)	33 (91.67)	37 (100.00)	0.073 ^2^
Current drinkers (*n*, %)	0 (0.00)	0 (0.00)	0.907 ^2^
Current smokers (*n*, %)	1 (2.78)	0 (0.00)	0.319 ^2^

Values are presented as mean ± SD or number (%). ^1^ Analyzed by independent *t*-test between the groups. ^2^ Analyzed by Chi-square test between the groups. ^3^ Analyzed by Wilcoxon rank sum test between the groups.

**Table 2:** Changes in body composition parameters assessed by dual-energy X-ray absorptiometry (DEXA) before and after 12-week intervention.

	GGE03 Group (*n* = 36)				Placebo Group (*n* = 37)				*p*-Value ^3^ (Between Group)
	Baseline	12 Week	Change Value	*p*-Value ^1^ (Within Group)	Baseline	12 Week	Change Value	*p*-Value ^1^ (Within Group)	
Body fat percentage (%)	32.16 ± 6.99	30.98 ± 7.59	−1.18 ± 3.12	0.028 ^2^	32.38 ± 7.13	32.75 ± 7.21	0.37 ± 2.29	0.090 ^2^	0.009 **
Body fat mass (lbs)	52.74 ± 15.52	50.74 ± 16.86	−2.01 ± 7.25	0.070 ^2^	52.39 ± 14.28	53.36 ± 15.19	0.98 ± 5.28	0.267	0.034 *
Lean body mass (lbs)	102.78 ± 12.27	105.56 ± 18.52	2.78 ± 16.39	0.900 ^2^	101.87 ± 15.72	101.07 ± 15.16	−0.80 ± 4.43	0.280	0.566

Values are presented as mean ± SD. ^1^ Analyzed by paired *t*-test between the baseline and 12 weeks within each group (weeks 0 vs. 12). ^2^ Analyzed by Wilcoxon signed rank test between the baseline and 12 weeks within each group (weeks 0 vs. 12). ^3^ Analyzed by Wilcoxon rank sum test for the change values between the groups (GGE03 vs. placebo). * *p* < 0.05, ** *p* < 0.01 vs. placebo group.

**Table 3:** Changes in anthropometric parameters before and after 12-week intervention.

	GGE03 Group (*n* = 36)				Placebo Group (*n* = 37)				*p*-Value ^3^ (Between Group)
	Baseline	12 Week	Change Value	*p*-Value ^1^ (Within Group)	Baseline	12 Week	Change Value	*p*-Value ^1^ (Within Group)	
Weight (kg)	75.00 ± 7.14	73.99 ± 7.26	−1.00 ± 1.88	0.007 ^2^	76.09 ± 7.57	76.71 ± 7.59	0.63 ± 1.92	0.090 ^2^	0.001 **
BMI (kg/m^2^)	26.56 ± 1.31	26.19 ± 1.47	−0.36 ± 0.67	0.006 ^2^	27.14 ± 1.63	27.38 ± 1.81	0.24 ± 0.68	0.046 ^2^	0.001 **
Waist circumference (cm)	82.19 ± 8.97	81.23 ± 8.38	−0.96 ± 2.06	0.008	81.73 ± 12.98	82.28 ± 12.86	0.55 ± 2.18	0.038 ^2^	0.001 **
Hip circumference (cm)	81.22 ± 8.77	80.09 ± 7.87	−1.13 ± 1.88	0.001	82.03 ± 12.41	82.30 ± 12.67	0.28 ± 2.07	0.172 ^2^	0.001 **
WHR	1.01 ± 0.04	1.01 ± 0.04	0.00 ± 0.02	0.396 ^2^	1.00 ± 0.04	1.00 ± 0.03	0.00 ± 0.02	0.189 ^2^	0.856

Values are presented as mean ± SD. ^1^ Analyzed by paired *t*-test between the baseline and 12 weeks within each group (weeks 0 vs. 12). ^2^ Analyzed by Wilcoxon signed rank test between the baseline and 12 weeks within each group (weeks 0 vs. 12). ^3^ Analyzed by Wilcoxon rank sum test for the change values between the groups (GGE03 vs. placebo). ** *p* < 0.01 vs. placebo group.

**Table 4:** Changes in serum lipid profiles and adiponectin levels before and after 12-week intervention.

	GGE03 Group (*n* = 36)				Placebo Group (*n* = 37)				*p*-Value ^3^ (Between Group)
	Baseline	12 Week	Change Value	*p*-Value ^1^ (Within Group)	Baseline	12 Week	Change Value	*p*-Value ^1^ (Within Group)	
Total cholesterol (mg/dL)	172.22 ± 24.85	169.28 ± 33.60	−2.94 ± 26.18	0.050 ^2^	175.08 ± 34.35	187.70 ± 36.04	12.62 ± 36.35	0.042	0.007 **
Triglyceride (mg/dL)	228.19 ± 146.81	216.47 ± 152.32	−11.72 ± 66.14	0.007 ^2^	223.49 ± 128.89	229.30 ± 147.43	5.81 ± 119.36	0.934 ^2^	0.043 *
LDL-cholesterol (mg/dL)	108.22 ± 23.01	108.36 ± 28.22	0.14 ± 22.37	0.330 ^2^	111.14 ± 29.92	121.57 ± 29.03	10.43 ± 30.20	0.043	0.055
HDL-cholesterol (mg/dL)	35.22 ± 6.35	36.72 ± 6.84	1.50 ± 5.46	0.109	35.32 ± 11.27	35.38 ± 7.33	0.05 ± 8.27	0.880 ^2^	0.397
Adiponectin (mg/dL)	6.32 ± 1.78	9.11 ± 7.78	2.79 ± 7.78	0.034 ^2^	6.36 ± 1.94	8.82 ± 11.05	2.46 ± 10.62	0.061 ^2^	0.712

Values are presented as mean ± SD. For adiponectin, one participant in the GGE03 group was excluded due to sample contamination. ^1^ Analyzed by paired *t*-test between the baseline and 12 weeks within each group (weeks 0 vs. 12). ^2^ Analyzed by Wilcoxon signed rank test between the baseline and 12 weeks within each group (weeks 0 vs. 12). ^3^ Analyzed by Wilcoxon rank sum test for the change values between the groups (GGE03 vs. placebo). * *p* < 0.05, ** *p* < 0.01 vs. placebo group.

**Table 5:** Changes in safety parameters before and after 12-week intervention.

		GGE03 Group (*n* = 36)				Placebo Group (*n* = 37)				*p*-Value ^3^ (Between Group)
		Baseline	12 Week	Change Value	*p*-Value ^1^ (Within Group)	Baseline	12 Week	Change Value	*p*-Value ^1^ (Within Group)	
CBC	Hemoglobin (g/dL)	14.07 ± 1.87	13.73 ± 2.04	−0.18 ± 2.65	0.675	13.74 ± 1.83	13.53 ± 2.01	−0.23 ± 2.23	0.515	0.929
	Hematocrit (%)	43.95 ± 4.76	43.28 ± 5.55	−0.22 ± 6.98	0.852	42.95 ± 4.53	42.82 ± 5.60	−0.18 ± 5.78	0.538 ^2^	1.000 ^4^
	WBC (10^9^/L)	6.95 ± 1.61	7.18 ± 3.32	0.38 ± 3.17	0.892 ^2^	6.89 ± 1.67	7.54 ± 2.05	0.71 ± 2.67	0.107	0.311 ^4^
	RBC (10^12^/L)	5.20 ± 0.64	5.13 ± 0.59	−0.03 ± 0.79	0.798	5.11 ± 0.52	5.05 ± 0.67	−0.07 ± 0.79	0.977 ^2^	0.884 ^4^
	Platelet (10^9^/L)	286.98 ± 65.57	319.84 ± 122.48	30.51 ± 139.80	0.365 ^2^	318.07 ± 74.60	317.38 ± 79.68	2.46 ± 102.09	0.881	0.473 ^4^
Biochemistry	AST (IU/L)	33.95 ± 16.65	33.38 ± 20.06	−0.76 ± 23.62	0.712 ^2^	29.57 ± 8.36	29.33 ± 16.26	−0.33 ± 18.46	0.105 ^2^	0.328 ^4^
	ALT (IU/L)	39.35 ± 21.81	35.16 ± 13.355	−3.97 ± 22.11	0.282	33.08 ± 16.76	26.15 ± 13.10	−7.08 ± 19.91	0.032	0.523
	ALP (IU/L)	99.33 ± 22.63	100.65 ± 33.40	0.11 ± 42.75	0.988	96.15 ± 21.13	92.13 ± 26.10	−4.13 ± 28.80	0.382	0.617
	GGT (U/L)	39.58 ± 24.39	47.30 ± 64.89	7.62 ± 65.57	0.759 ^2^	34.73 ± 23.23	32.08 ± 24.39	−3.15 ± 37.15	0.497 ^2^	0.473 ^4^
	Albumin (g/dL)	4.50 ± 0.22	4.46 ± 0.42	−0.03 ± 0.42	0.696	4.47 ± 0.52	4.39 ± 0.41	−0.08 ± 0.55	0.052 ^2^	0.374 ^4^
	Total protein (g/dL)	7.34 ± 0.41	7.47 ± 0.51	0.14 ± 0.66	0.192	7.38 ± 0.38	7.39 ± 0.48	0.00 ± 0.53	0.960	0.285
	Total bilirubin (mg/dL)	0.66 ± 0.24	0.60 ± 0.27	−0.05 ± 0.36	0.449	0.57 ± 0.27	0.53 ± 0.25	−0.02 ± 0.36	0.682	0.794
	Glucose (mg/dL)	97.08 ± 13.65	115.95 ± 46.92	18.62 ± 45.20	0.034 ^2^	94.95 ± 13.61	105.79 ± 38.56	7.87 ± 43.05	0.783 ^2^	0.192 ^4^
	BUN (mg/dL)	9.22 ± 2.74	12.46 ± 11.33	3.52 ± 11.41	0.044 ^2^	8.94 ± 2.29	12.74 ± 7.31	3.81 ± 7.24	<0.001 ^2^	0.196 ^4^
	Creatinine (mg/dL)	0.91 ± 0.15	1.04 ± 0.73	0.15 ± 0.73	0.993 ^2^	0.90 ± 0.14	0.89 ± 0.50	−0.01 ± 0.45	0.033 ^2^	0.177 ^4^
	Uric Acid (mg/dL)	6.17 ± 1.36	5.76 ± 1.68	−0.32 ± 1.89	0.097 ^2^	6.34 ± 1.39	5.13 ± 1.53	−1.24 ± 1.90	0.001 ^2^	0.201 ^4^
Urinalysis	pH	5.83 ± 0.72	5.96 ± 0.64	0.08 ± 0.92	0.594	5.95 ± 0.74	5.88 ± 0.57	−0.03 ± 0.92	0.717 ^2^	0.475 ^4^
	Specific gravity	1.01 ± 0.01	1.01 ± 0.01	0.00 ± 0.01	0.369	1.01 ± 0.01	1.02 ± 0.01	0.00 ± 0.01	0.009	0.315
Vital signs	Systolic blood pressure (mmHg)	116.22 ± 3.99	117.81 ± 4.53	1.78 ± 6.21	0.089	115.08 ± 5.75	117.21 ± 3.87	2.10 ± 6.45	0.056 ^2^	0.830 ^4^
	Diastolic blood pressure (mmHg)	71.50 ± 5.87	74.65 ± 5.98	3.14 ± 9.42	0.050	72.65 ± 5.68	74.26 ± 4.59	1.74 ± 7.11	0.174 ^2^	0.338 ^4^
	Pulse (beats/min)	83.62 ± 7.60	80.62 ± 8.27	−3.19 ± 11.70	0.106	82.75 ± 7.37	83.28 ± 7.29	0.72 ± 10.76	0.679	0.135

Values are presented as mean ± SD. ^1^ Analyzed by paired *t*-test between the baseline and 12 weeks within each group (weeks 0 vs. 12). ^2^ Analyzed by Wilcoxon signed rank test between the baseline and 12 weeks within each group (weeks 0 vs. 12). ^3^ Analyzed by independent *t*-test for the change values between the groups (GGE03 vs. placebo). ^4^ Analyzed by Wilcoxon rank sum test for the change values between the groups (GGE03 vs. placebo). **Abbreviations**: CBC, complete blood count; WBC, white blood cell; RBC, red blood cell; AST, aspartate aminotransferase; ALT, alanine aminotransferase; ALP, alkaline phosphatase; GGT, γ-glutamyl transferase; BUN, blood urea nitrogen.

## Data Availability

The datasets generated and analyzed during the current study are available from the corresponding author on reasonable request. The data are not publicly available due to privacy and ethical restrictions.

## References

[1] Wen X., Zhang B., Wu B., Xiao H., Li Z., Li R., Xu X., Li T. (2022). Signaling pathways in obesity: Mechanisms and therapeutic interventions. Signal Transduct. Target. Ther..

[2] Gregg E.W., Shaw J.E. (2017). Global Health Effects of Overweight and Obesity. N. Engl. J. Med..

[3] Boutari C., Mantzoros C.S. (2022). A 2022 update on the epidemiology of obesity and a call to action: As its twin COVID-19 pandemic appears to be receding, the obesity and dysmetabolism pandemic continues to rage on. Metabolism.

[4] Phelps N.H., Singleton R.K., Zhou B., Heap R.A., Mishra A., Bennett J.E., Paciorek C.J., Lhoste V.P.F., Carrillo-Larco R.M., Stevens G.A. (2024). Worldwide trends in underweight and obesity from 1990 to 2022: A pooled analysis of 3663 population-representative studies with 222 million children, adolescents, and adults. Lancet.

[5] Seidell J.C., Halberstadt J. (2015). The global burden of obesity and the challenges of prevention. Ann. Nutr. Metab..

[6] Kim D.H., Yang B.S. (2006). Molecular mechanisms underlying the link of obesity to chronic diseases. Korean J. Obes..

[7] Vuik S., Lerouge A., Guillemette Y., Feigl A., Aldea A. (2019). The Economic Burden of Obesity.

[8] Masood B., Moorthy M. (2023). Causes of obesity: A review. Clin. Med..

[9] Bleich S.N., Cutler D., Murray C., Adams A. (2008). Why Is the Developed World Obese?. Annu. Rev. Public Health.

[10] Schwartz M.W., Seeley R.J., Zeltser L.M., Drewnowski A., Ravussin E., Redman L.M., Leibel R.L. (2017). Obesity Pathogenesis: An Endocrine Society Scientific Statement. Endocr. Rev..

[11] Albuquerque D., Nóbrega C., Manco L., Padez C. (2017). The contribution of genetics and environment to obesity. Br. Med. Bull..

[12] Swinburn B.A., Sacks G., Hall K.D., McPherson K., Finegood D.T., Moodie M.L., Gortmaker S.L. (2011). The global obesity pandemic: Shaped by global drivers and local environments. Lancet.

[13] Lin X., Li H. (2021). Obesity: Epidemiology, Pathophysiology, and Therapeutics. Front. Endocrinol..

[14] Pi-Sunyer X. (2009). The medical risks of obesity. Postgrad. Med..

[15] Chakhtoura M., Haber R., Ghezzawi M., Rhayem C., Tcheroyan R., Mantzoros C.S. (2023). Pharmacotherapy of obesity: An update on the available medications and drugs under investigation. EClinicalMedicine.

[16] Son J.W., Kim S. (2020). Comprehensive review of current and upcoming anti-obesity drugs. Diabetes Metab. J..

[17] Khera R., Murad M.H., Chandar A.K., Dulai P.S., Wang Z., Prokop L.J., Loomba R., Camilleri M., Singh S. (2016). Association of pharmacological treatments for obesity with weight loss and adverse events: A systematic review and meta-analysis. Jama.

[18] Shaik Mohamed Sayed U.F., Moshawih S., Goh H.P., Kifli N., Gupta G., Singh S.K., Chellappan D.K., Dua K., Hermansyah A., Ser H.L. (2023). Natural products as novel anti-obesity agents: Insights into mechanisms of action and potential for therapeutic management. Front. Pharmacol..

[19] Patil B.S., Patil J.K., Chaudhari H.S., Patil B.S. (2025). Oxidative Stress, Inflammation, and Obesity: Insights into Mechanism and Therapeutic Targets. Proceedings.

[20] Connaughton R.M., McMorrow A.M., McGillicuddy F.C., Lithander F.E., Roche H.M. (2016). Impact of anti-inflammatory nutrients on obesity-associated metabolic-inflammation from childhood through to adulthood. Proc. Nutr. Soc..

[21] Jiang S., Liu H., Li C. (2021). Dietary regulation of oxidative stress in chronic metabolic diseases. Foods.

[22] Ayustaningwarno F., Anjani G., Ayu A.M., Fogliano V. (2024). A critical review of Ginger’s (*Zingiber officinale*) antioxidant, anti-inflammatory, and immunomodulatory activities. Front. Nutr..

[23] Wang J., Ke W., Bao R., Hu X., Chen F. (2017). Beneficial effects of ginger Zingiber officinale Roscoe on obesity and metabolic syndrome: A review. Ann. N. Y. Acad. Sci..

[24] Preciado-Ortiz M.E., Gembe-Olivarez G., Martínez-López E., Rivera-Valdés J.J. (2025). Immunometabolic effects of ginger (*Zingiber officinale* Roscoe) supplementation in obesity: A comprehensive review. Molecules.

[25] Bartley J.P., Jacobs A.L. (2000). Effects of drying on flavour compounds in Australian-grown ginger (*Zingiber officinale*). J. Sci. Food Agric..

[26] Fuhrman B., Rosenblat M., Hayek T., Coleman R., Aviram M. (2000). Ginger extract consumption reduces plasma cholesterol, inhibits LDL oxidation and attenuates development of atherosclerosis in atherosclerotic, apolipoprotein E-deficient mice. J. Nutr..

[27] Mishra R.K., Kumar A., Kumar A. (2012). Pharmacological activity of Zingiber officinale. Int. J. Pharm. Chem. Sci..

[28] Seo S.H., Fang F., Kang I. (2021). Ginger (*Zingiber officinale*) attenuates obesity and adipose tissue remodeling in high-fat diet-fed C57BL/6 mice. Int. J. Environ. Res. Public Health.

[29] Mao Q.-Q., Xu X.-Y., Cao S.-Y., Gan R.-Y., Corke H., Beta T., Li H.-B. (2019). Bioactive compounds and bioactivities of ginger (*Zingiber officinale* Roscoe). Foods.

[30] Nishidono Y., Tanaka K. (2023). Effect of drying and processing on diterpenes and other chemical constituents of ginger. J. Nat. Med..

[31] Abdo M.T., El-Ahmady S.H., Gad H.A. (2021). Quality control and long-term stability study of ginger from different geographical origins using chemometrics. J. Sci. Food Agric..

[32] Tóth B., Horváth A., Laczkovich O.J., Biró Z.D., Matuz M., Csupor D. (2024). Storage Conditions Influence the Quality of Ginger–A Stability Study Inspired by Clinical Trials. Planta Medica.

[33] Cheng X.-L., Liu Q., Peng Y.-B., Qi L.-W., Li P. (2011). Steamed ginger (*Zingiber officinale*): Changed chemical profile and increased anticancer potential. Food Chem..

[34] Choi Y.J., Jung J.I., Lim S., Kim C.S., Park D.W., Lee S.H., Hur N., Bae J., Lee J.K., Kim E.J. (2025). Steamed Ginger Extract (GGE03) Attenuates Obesity and Improves Metabolic Parameters in Association with AMPK Activation and Lipid Metabolism Regulation in High-Fat Diet-Induced Obese Mice. Int. J. Mol. Sci..

[35] Nam Y.H., Hong B.N., Rodriguez I., Park M.S., Jeong S.Y., Lee Y.-G., Shim J.H., Yasmin T., Kim N.W., Koo Y.T. (2020). Steamed ginger may enhance insulin secretion through KATP channel closure in pancreatic β-cells potentially by increasing 1-dehydro-6-gingerdione content. Nutrients.

[36] Shin J.-K., Park J.H., Kim K.S., Kang T.H., Kim H.S. (2020). Antiulcer Activity of Steamed Ginger Extract against Ethanol/HCl-Induced Gastric Mucosal Injury in Rats. Molecules.

[37] Song M.Y., Lee D.Y., Park S.Y., Seo S.A., Hwang J.S., Heo S.H., Kim E.H. (2021). Steamed Ginger Extract Exerts Anti-inflammatory Effects in Helicobacter pylori-infected Gastric Epithelial Cells through Inhibition of NF-κB. J. Cancer Prev..

[38] Baek H.-I., Ha N.-R., Kim C., Im T.J., Kim Y.Y., Hwang S.H., Bae J.W. (2025). Efficacy and safety of steamed ginger extract for gastric health: A randomized, double-blind, placebo-controlled multi-center clinical trial. Food Funct..

[39] Baek H.-I., Shen L., Ha K.-C., Park Y.K., Kim C.S., Kwon J.E., Park S.J. (2024). Effectiveness and safety of steamed ginger extract on mild osteoarthritis: A randomized, double-blind, placebo-controlled clinical trial. Food Funct..

[40] Park S.-H., Jung S.-J., Choi E.-K., Ha K.-C., Baek H.-I., Park Y.-K., Han K.-H., Jeong S.-Y., Oh J.-H., Cha Y.-S. (2020). The effects of steamed ginger ethanolic extract on weight and body fat loss: A randomized, double-blind, placebo-controlled clinical trial. Food Sci. Biotechnol..

[41] Lee Y.-G., Lee S.R., Baek H.J., Kwon J.E., Baek N.-I., Kang T.H., Kim H., Kang S.C. (2024). The effects of body fat reduction through the metabolic control of steam-processed ginger extract in high-fat-diet-fed mice. Int. J. Mol. Sci..

[42] Kim H.-J., Kim B., Mun E.-G., Jeong S.-Y., Cha Y.-S. (2018). The antioxidant activity of steamed ginger and its protective effects on obesity induced by high-fat diet in C57BL/6J mice. Nutr. Res. Pract..

[43] Kim B., Kim H.-J., Cha Y.-S. (2021). The protective effects of steamed ginger on adipogenesis in 3T3-L1 cells and adiposity in diet-induced obese mice. Nutr. Res. Pract..

[44] Lee G.-H., Peng C., Jeong S.-Y., Park S.-A., Lee H.-Y., Hoang T.-H., Kim J., Chae H.-J. (2021). Ginger extract controls mTOR-SREBP1-ER stress-mitochondria dysfunction through AMPK activation in obesity model. J. Funct. Foods.

[45] Aggarwal B.B. (2010). Targeting inflammation-induced obesity and metabolic diseases by curcumin and other nutraceuticals. Annu. Rev. Nutr..

[46] Shehzad A., Ha T., Subhan F., Lee Y.S. (2011). New mechanisms and the anti-inflammatory role of curcumin in obesity and obesity-related metabolic diseases. Eur. J. Nutr..

[47] Anand P., Kunnumakkara A.B., Newman R.A., Aggarwal B.B. (2007). Bioavailability of curcumin: Problems and promises. Mol. Pharm..

[48] Dei Cas M., Ghidoni R. (2019). Dietary curcumin: Correlation between bioavailability and health potential. Nutrients.

[49] Cho Y.-G., Jung J.-H., Kang J.-H., Kwon J.S., Yu S.P., Baik T.G. (2017). Effect of a herbal extract powder (YY-312) from Imperata cylindrica Beauvois, Citrus unshiu Markovich, and Evodia officinalis Dode on body fat mass in overweight adults: A 12-week, randomized, double-blind, placebo-controlled, parallel-group clinical trial. BMC Complement. Altern. Med..

[50] Rafieipour N., Gharbi N., Rahimi H., Kohansal A., Sadeghi-Dehsahraei H., Fadaei M., Tahmasebi M., Momeni S.A., Ostovar N., Ahmadi M. (2024). Ginger intervention on body weight and body composition in adults: A GRADE-assessed systematic review and dose-response meta-analysis of 27 randomized controlled trials. Nutr. Rev..

[51] Rjabi S., Seyedhatami S.S., Makhtoomi M., Ahmadi M.R., Alimohamadi S., Aliabadi E., Talakesh S., Nouri M., Zamani B., Askarpour M. (2025). Impact of ginger supplementation on obesity indices and Adipokine profiles in adults: A GRADE-based systematic review and dose-response meta-analysis of randomized controlled trials. Complement. Ther. Med..

[52] Magkos F., Fraterrigo G., Yoshino J., Luecking C., Kirbach K., Kelly S.C., de Las Fuentes L., He S., Okunade A.L., Patterson B.W. (2016). Effects of moderate and subsequent progressive weight loss on metabolic function and adipose tissue biology in humans with obesity. Cell Metab..

[53] Rothberg A.E., McEwen L.N., Kraftson A.T., Ajluni N., Fowler C.E., Nay C.K., Miller N.M., Burant C.F., Herman W.H. (2017). Impact of weight loss on waist circumference and the components of the metabolic syndrome. BMJ Open Diabetes Res. Care.

[54] Zhang X., Wang Y., Li Y., Gui J., Mei Y., Yang X., Liu H., Guo L.-L., Li J., Lei Y. (2024). Four-years change of BMI and waist circumference are associated with metabolic syndrome in middle-aged and elderly Chinese. Sci. Rep..

[55] Ebrahimzadeh Attari V., Ostadrahimi A., Asghari Jafarabadi M., Mehralizadeh S., Mahluji S. (2016). Changes of serum adipocytokines and body weight following Zingiber officinale supplementation in obese women: A RCT. Eur. J. Nutr..

[56] Maharlouei N., Tabrizi R., Lankarani K.B., Rezaianzadeh A., Akbari M., Kolahdooz F., Rahimi M., Keneshlou F., Asemi Z. (2019). The effects of ginger intake on weight loss and metabolic profiles among overweight and obese subjects: A systematic review and meta-analysis of randomized controlled trials. Crit. Rev. Food Sci. Nutr..

[57] Ormazabal V., Nair S., Elfeky O., Aguayo C., Salomon C., Zuñiga F.A. (2018). Association between insulin resistance and the development of cardiovascular disease. Cardiovasc. Diabetol..

[58] Wing R.R., Lang W., Wadden T.A., Safford M., Knowler W.C., Bertoni A.G., Hill J.O., Brancati F.L., Peters A., Wagenknecht L. (2011). Benefits of modest weight loss in improving cardiovascular risk factors in overweight and obese individuals with type 2 diabetes. Diabetes Care.

[59] Lavie C.J., Milani R.V., Ventura H.O. (2009). Obesity and cardiovascular disease: Risk factor, paradox, and impact of weight loss. J. Am. Coll. Cardiol..

[60] Ross R., Neeland I.J., Yamashita S., Shai I., Seidell J., Magni P., Santos R.D., Arsenault B., Cuevas A., Hu F.B. (2020). Waist circumference as a vital sign in clinical practice: A Consensus Statement from the IAS and ICCR Working Group on Visceral Obesity. Nat. Rev. Endocrinol..

[61] Després J.-P. (2012). Body fat distribution and risk of cardiovascular disease: An update. Circulation.

[62] Kuk J.L., Katzmarzyk P.T., Nichaman M.Z., Church T.S., Blair S.N., Ross R. (2006). Visceral fat is an independent predictor of all-cause mortality in men. Obesity.

[63] Bazzocchi A., Ponti F., Albisinni U., Battista G., Guglielmi G. (2016). DXA: Technical aspects and application. Eur. J. Radiol..

[64] Lee S.Y., Ahn S., Kim Y.J., Ji M.J., Kim K.M., Choi S.H., Jang H.C., Lim S. (2018). Comparison between dual-energy X-ray absorptiometry and bioelectrical impedance analyses for accuracy in measuring whole body muscle mass and appendicular skeletal muscle mass. Nutrients.

[65] Willoughby D., Hewlings S., Kalman D. (2018). Body composition changes in weight loss: Strategies and supplementation for maintaining lean body mass, a brief review. Nutrients.

[66] Johannsen D.L., Knuth N.D., Huizenga R., Rood J.C., Ravussin E., Hall K.D. (2012). Metabolic slowing with massive weight loss despite preservation of fat-free mass. J. Clin. Endocrinol. Metab..

[67] Grundy S.M., Brewer H.B., Cleeman J.I., Smith S.C., Lenfant C. (2004). Definition of metabolic syndrome: Report of the National Heart, Lung, and Blood Institute/American Heart Association conference on scientific issues related to definition. Circulation.

[68] O’Hara M., Kiefer D., Farrell K., Kemper K. (1998). A review of 12 commonly used medicinal herbs. Arch. Fam. Med..

[69] Okonta J., Uboh M., Obonga W. (2008). Herb-drug interaction: A case study of effect of ginger on the pharmacokinetic of metronidazole in rabbit. Indian J. Pharm. Sci..

[70] Weidner M.S., Sigwart K. (2000). The safety of a ginger extract in the rat. J. Ethnopharmacol..

[71] Wang T., Huang T., Zheng Y., Rood J., Bray G.A., Sacks F.M., Qi L. (2016). Genetic variation of fasting glucose and changes in glycemia in response to 2-year weight-loss diet intervention: The POUNDS LOST trial. Int. J. Obes..

[72] Metcalfe R.S., Gurd B.J., Vollaard N.B. (2023). Exploring interindividual differences in fasting and postprandial insulin sensitivity adaptations in response to sprint interval exercise training. Eur. J. Sport Sci..

[73] Utzschneider K.M., Johnson T.N., Breymeyer K.L., Bettcher L., Raftery D., Newton K.M., Neuhouser M.L. (2020). Small changes in glucose variability induced by low and high glycemic index diets are not associated with changes in β-cell function in adults with pre-diabetes. J. Diabetes Its Complicat..

